# Cabozantinib in neuroendocrine tumors: tackling drug activity and resistance mechanisms

**DOI:** 10.1530/ERC-23-0232

**Published:** 2023-10-18

**Authors:** Chiara Alessandra Cella, Riccardo Cazzoli, Nicola Fazio, Giuseppina De Petro, Germano Gaudenzi, Silvia Carra, Mauro Romanenghi, Francesca Spada, Ilaria Grossi, Isabella Pallavicini, Saverio Minucci, Giovanni Vitale

**Affiliations:** 1 Division of Gastrointestinal Medical Oncology and Neuroendocrine Tumors, European Institute of Oncology, IEO, IRCCS, Milan, Italy; 2 Department of Molecular and Translational Medicine, Division of Biology and Genetics, University of Brescia, Brescia, Italy; 3 Department of Experimental Oncology, European Institute of Oncology, IEO, IRCCS, Milan, Italy; 4 Metal Targeted Therapy & Immunology lab, Childrens’ cancer institute, Sydney, NSW, Australia; 5 Laboratory of Geriatric and Oncologic Neuroendocrinology Research, IRCCS, Istituto Auxologico Italiano, Milan, Italy; 6 Laboratory of Endocrine and Metabolic Research, IRCCS, Istituto Auxologico Italiano, Milan, Italy; 7 Department of Oncology and Hemato-Oncology, University of Milan, Milan, Italy; 8 Department of Medical Biotechnology and Translational Medicine, University of Milan, Milan, Italy

**Keywords:** molecular biology, cancer, tumor angiogenesis, zebrafish model, mouse models

## Abstract

Neuroendocrine tumors (NETs) are highly vascularized malignancies in which angiogenesis may entail cell proliferation and survival. Among the emerging compounds with antivascular properties, cabozantinib (CAB) appeared promising. We analyzed the antitumor activity of CAB against NETs utilizing *in vitro* and *in vivo* models. For cell cultures, we used BON-1, NCI-H727 and NCI-H720 cell lines. Cell viability was assessed by manual count coupled with quantification of cell death, performed through fluorescence-activated cell sorting analysis as propidium iodide exclusion assay. In addition, we investigated the modulation of the antiapoptotic myeloid cell leukemia 1 protein under CAB exposure, as a putative adaptive pro-survival mechanism, and compared the responses with sunitinib. The activity of CAB was also tested in mouse and zebrafish xenograft tumor models. Cabozantinib showed a dose-dependent and time-dependent effect on cell viability and proliferation in human NET cultures, besides a halting of cell cycle progression for endoduplication, never reported for other tyrosine kinase inhibitors. In a transplantable zebrafish model, CAB drastically inhibited NET-induced angiogenesis and migration of implanted cells through the embryo body. CAB showed encouraging activity in NETs, both *in vitro* and *in vivo* models. On this basis, we envisage future research to further investigate along these promising lines.

## Introduction

Neuroendocrine neoplasms (NENs) constitute a highly heterogeneous spectrum of malignancies. In the majority of cases, they appear as well-differentiated or a low/intermediate grade of malignancy and are termed neuroendocrine tumors (NETs), to be distinguished from neuroendocrine carcinomas (NECs) which are the poorly differentiated or high-grade forms. NETs have a variety of biological and clinical behaviors and are known to be highly vascularized. Accumulating knowledge about the molecular landscape of NETs led to the hypothesis that a deregulation of the mTOR pathway alongside an aberrant angiogenesis activation may be indicated as a crucial determinant for stimulating cell proliferation and survival (Casanovas *et al.* 2005, Couvelard *et al.* 2005). Specifically, the blockade of the vascular endothelial growth factor (VEGF) pathway exerts multiple downstream effects, implying inhibitory effects on vascular pruning other than angiogenesis signaling, thereby enhancing the hypoxia pathway and subsequently the mesenchymal–epithelial transition (MET) (Rapisarda *et al.* 2009, Villaume *et al.* 2010). The upregulation of mesenchymal–epithelial transition factor (c-MET), which is known to be involved in the invasive and metastatic behavior of tumor cells under hypoxia conditions, has also been reported in RIP-Tag2 mice after a chronic exposure to antivascular drugs (Sennino *et al.* 2012). Accordingly, the concomitant inhibition of VEGF and MET signaling has been determined as a key point for overcoming the resistance to a prior antiangiogenic treatment, such as sunitinib (SUN), the only multi-targeted tyrosine kinase inhibitor (TKI) approved for pancreatic NETs (Raymond *et al.* 2011, Yao *et al.* 2011). Furthermore, the adaptive pro-survival responses that NET cells activate to maintain their viability and to tolerate the effects of TKIs are still under investigation. Some authors have focused their attention on the modulation of mTOR signaling and the level of the antiapoptotic protein myeloid cell leukemia 1 (MCL-1) as a crucial determinant of cell survival and resistance to anticancer agents (Elgendy *et al.* 2016). In a previous study, Elgendy and colleagues found that MCL-1 stands out as a unique member of the B-cell lymphoma 2 (BCL2) family with a short half-life and a complex regulation which can be finely tuned in response to different cellular stresses, under SUN exposure (Elgendy *et al.* 2016). Overall, aiming to tackle the multifactorial genesis of acquired resistance, several novel TKIs, mainly with antiangiogenic properties, have been investigated over the last decade in NETs, such as pazopanib (Grande *et al.* 2015), sorafenib (Chan *et al.* 2013), axitinib (Strosberg *et al.* 2016), surufatinib (Xu *et al.* 2020*a*, *b*), lenvatinib (Capdevila *et al.* 2021) and cabozantinib (CAB) (Yakes *et al.* 2011) in addition to the monoclonal antibody bevacizumab (Chan *et al.* 2012). Although none of them have been granted immediate approval in NETs neither by the FDA nor by the EMA at the time of writing, CAB has been the subject of increasing attention. CAB works as an ATP-competitive inhibitor of VEGFR2 and MET kinases, besides other targets such as RET, AXL and FLT3. Preclinical evidence clarified that the dual blockade of MET and VEGF signaling in RIP-Tag2 mice may reduce the invasive and metastatic capabilities of pancreatic NETs (paNET) cells with a synergistic effect, thereby providing a strong rationale for using CAB in this setting (Sennino *et al.* 2012). A few years later, Reuther and colleagues corroborated these findings proving that the multi-tyrosine kinase inhibitors, CAB and tivantinib, exerted promising antitumor and antimigratory effects in NET cells, while the highly specific c-MET inhibitor INC280 had no inhibitory effect on cell viability and migration suggesting that c-MET inhibition alone is not sufficient to exert direct antitumoral effects in NET models (Reuther *et al.* 2016).

Consistently with these data, and due to its ability to concomitantly inhibit different key pathways, CAB may presumably exert its antitumor efficacy by inhibiting angiogenesis and decreasing the invasive capabilities of tumor cells rather than affecting cell proliferation. However, preclinical and clinical studies exploring the pleiotropic activity of CAB in NETs should be further encouraged.

Over the last decade, the use of zebrafish (*Daniorerio*) in biomedical research has been growing exponentially with relevant applications in studying human diseases and cancer modeling (Löhr & Hammerschmidt 2011). Vitale and colleagues have developed a reliable zebrafish model to investigate tumor-induced angiogenesis and metastatic behavior in NENs, based on the injection of human NET cell lines and patient-derived xenograft in the proximity of the developing sub-intestinal vessels (SIVs) in zebrafish embryos (Vitale *et al.* 2014, Gaudenzi *et al.* 2017, Carra & Gaudenzi 2020). Basically, the appeal of the zebrafish xenograft resides in the possibility of overcoming certain drawbacks of murine xenografts, such as a large number of tumor cells needed (about 1 million), the long time required (from several weeks to months) to have a visible tumor implant, the need for immunosuppressed animals to avoid transplant rejection and the great difficulty of generating mouse xenotransplant models able to metastasize. Furthermore, embryos are readily permeable to small molecules dissolved in their culture media, thereby making the zebrafish/xenograft model a worthwhile tool to test *in vivo* the antitumor activity of several antiangiogenic compounds.

The aim of this study is to investigate the activity of CAB in different NET models. Additionally, we examined the adaptive pro-survival responses that tumor cells exploit for maintaining their viability and tolerating the cytotoxic effects triggered by CAB, focusing on the modulation of the antiapoptotic MCL-1 protein. We then further compared the relevance of those adaptive responses to intrinsic, as well as acquired, resistance of cancer cells to SUN, the only antiangiogenic TKI approved in NET. We finally applied an attractive method for testing *in vivo* the antitumor activity of CAB in a NET transplantable zebrafish model.

## Materials and methods

### Cell cultures

For cell culture and reagents, the following human NEN cell lines were used:

- BON-1 (tumor cells derived from peripancreatic lymph node metastasis of paNENs)
- NCI-H727 (tumor cells derived from typical pulmonary carcinoids)
- NCI-H720 (tumor cells derived from atypical pulmonary carcinoids)

Cell lines were obtained from Dr Valeria Giandomenico, Department of Medical Sciences, Endocrine Tumor Biology, Uppsala University, Uppsala, Sweden. Cell lines are tested by our cell culture facility to confirm identity by the use of STR profiling.

BON-1 and NCI-H727 cells were cultured in DMEM (ECM0103L Euroclone), supplemented with 10% South American fetal bovine serum and 1% of glutamine and 1% of sodium pyruvate. NCI-H720 cells were cultured in RPMI1640 (ECM2001L Euroclone) supplemented with 10% of North American fetal bovine serum, 1% of glutamine.

We tested the drug at different concentrations (ranging from 0.5 to 10 µM, which is higher than a pharmacologically relevant concentration, based on the known pharmacokinetic parameters of the drug) on four human NET tumor cell lines of interest.

### Quantification of cell proliferation

Cell viability was assessed by manual count coupled with quantification of cell death, performed through fluorescence-activated cell sorting (FACS) analysis as a propidium iodide (PI) exclusion assay (MACSquant, Miltenyi Biotec).

Cell viability assay was performed with CellTiter-Glo® Luminescent Cell Viability Assay (G7570 Promega). 2500 cell/well in adhesion (BON-1 and H727) and 5000 cell/well in suspension (H720) were plated into 96-flat-bottom well plates, tissue culture treated (Costar 3917) and grown for 24 h. Cells were then treated with either 0.1% DMSO or varying concentrations (0.01–10 µM) of the test compounds up to 72 or 144 h, according to the manufacturer protocol. Two wells were used for each cell line and drug concentration. Reading was performed by Glomax Explorer (Promega). Cells were counted by trypan blue exclusion. Data were plotted as a fraction of viability vs untreated (*y*-axis, relative to NT) at increasing concentrations of CAB (*x*-axis). CAB was provided by Ipsen Company.

### Quantification of cell death

Quantification of cell death was performed through FACS analysis (cell cycle and apoptosis). Cells were fixed in ethanol and then PI was incorporated overnight at 4°C according to the protocol. FACS analysis was performed with FACSCelesta (BD) performed by the Flow Cytometry Unit (IEO Campus) after PI incorporation. CAB was purchased by Selleck (code S1119).

### Immunoblotting

Western blot assays were performed using the following antibodies: MCL-1 MoAb used at a dilution of 1:1000 (4572 Cell Signaling), vinculin MoAb (Sigma-Aldrich, dilution of 1:10,000), C-MET (Santa Cruz, 1:1000), P-MET (Cell Signaling, 1:1000), 4EBP (Cell Signaling, 1:1000), P-4EBP (Cell Signalling, 1:1000), S6 (Cell Signaling, 1:1000) and P-S6 (Cell Signaling, 1:1000). Antibodies were purchased from the indicated sources.

Cells were seeded (1.5 × 10^6^ in adhesion and 4 × 10^6^ in suspension) and resuspended after drug treatment in a urea buffer; then protein concentration was determined by a protein assay kit (Bio-Rad). Proteins (80 µg) were separated in 12% SDS–polyacrylamide gels, transferred onto a nitrocellulose membrane and probed against MCL-1 MoAb. The secondary antibody was mouse IgGκ-binding protein HRP. Proteins were visualized with Clarity Western ECL and analyzed with Chemidoc (Bio-Rad).

### Zebrafish care and maintenance

Adult zebrafish was maintained according to National (Italian Legislative Decree 26/2014) and European laws (2010/63/EU and 86/609/EEC), which regulate experiments on live animals.

Ethical approval for experimentation was not required since tumor xenografts will be performed only on zebrafish embryos and larvae, within 5 days post fertilization. According to the description of OECD TG236, zebrafish within this time window is generally not considered as being capable of independent feeding; thus, this is not considered animal experimentation. This is confirmed by the Commission Implementing Decision 2012/707/EU (EU 2012b) on a common format on the collection of information on the use of animals for scientific purposes.

Embryos, collected by natural spawning, were staged and raised at 28°C in ﬁsh water (0.1 g/L NaHCO_3_, 0.1 g/L Instant Ocean, 0.192 g/L CaSO_4_.2H_2_O) containing 0.003% PTU (1-phenyl-2-thiourea; Sigma-Aldrich) to prevent pigmentation and 0.01% methylene blue to prevent fungal growth.

### Zebrafish xenograft model

The antitumor activity of CAB was analyzed in a transplantable zebrafish model. Four human NET cell lines (BON-1, NCI-H727 and NCI-H720 – tumor cells derived from paNENs) were implanted in 48 h post-fertilization (hpf) *Tg(ﬂi1a:EGFP)^y1^* zebrafish embryos. This transgenic line expresses enhanced green fluorescent protein (EGFP) under the control of the endothelial fli1a promoter, providing a live visual marker for vascular development. Before the implantation, tumor cells were stained with a red fluorescent cell tracker dye in order to attain the optimal visualization using epifluorescence microscopy. After the resuspension in PBS, about 500 NET cells were injected into the subperidermal space, between the periderm and the yolk syncytial layer, close to the subintestinal vein (SIV) plexus. Due to the permeability of embryonic tissue to small molecules, CAB was directly dissolved into the medium soon after the implantation of NET cells. Two concentrations (0.25 and 2.5 µM) of CAB were selected on the basis of preliminary tests on the viability of *Tg(ﬂi1a:EGFP)^y1^* embryos without tumor xenograft. As a control of the pharmacological treatment, we considered embryos treated with DMSO, the vehicle in which CAB was dissolved.

### Evaluation of angiogenic and migratory response

Starting from 24 hpi, both the pro-angiogenic and migratory responses were monitored *in vivo* by means of epifluorescence (Leica M205FA equipped with a Leica DFC450C digital camera; Leica) microscope in all embryos. After NEN cell engraftment, tumor-induced angiogenesis was considered positive when SIV development began, and endothelial cells sprouted from the SIV and/or common cardinal vein (CCV) toward the xenograft area. As an arbitrary unit of tumor-induced angiogenesis, the EGFP area corresponding to endothelial structures that sprouted from the SIV plexus was quantified using Fiji software. In particular, we selected a region of interest (ROI) in the area surrounding the graft of each embryo, in which only tumor-induced endothelial structures were included and normal developmental vessels were not considered. Afterward, we set the same threshold for the EGFP channel in each embryo, in order to exclude the background signal. Thus, we limited to threshold the calculation of the area in each selected ROI. Moreover, we considered the situation as one of ‘active migration’ if the labeled NET cells were identified outside the yolk sac region in particular in the tail region. At 48 hpi, the presence of tumor cell clusters far from the injection site was quantified by the ‘Analyze Particle’ plugin of the Fiji software in embryos. Data from both tumor-induced angiogenesis and invasiveness analyses were normalized against the mean of the lowest values, arbitrarily set to 1.0. All experiments were performed at least three times, considering at least 20 embryos in each experimental group. Statistical differences among groups were evaluated by the analysis of variance (ANOVA) test together with a *post hoc* Tukey’s multiple comparison test. A *P*-value < 0.05 was considered statistically significant. The values reported in the graphs represent the mean ± s.e.m .For statistical analysis, GraphPad Prism 5.0 was used (GraphPad Software Inc.).

### Mouse xenograft model

The effect of CAB was tested in a xenograft tumor model. We inoculated human BON1 cells subcutaneously on mice flanks (5 × 10^6^ cells in 200 μL of sterile PBS) and divided CD1 nude mice (8–10 weeks old, Charles River) into three groups (six mice/group). We treated the mice by oral gavage with vehicle (4% DMSO in sterile water) or two doses (40 mg/kg (10 μL/g mouse) and 60 mg/kg (15 μL/g mouse) in 4% DMSO in sterile water) of CAB. Treatment was repeated every day for 3 weeks, at the end of the last treatment, mice were euthanized by CO_2_ asphyxiation and autopsied. Tumor size was measured by calipers and mice weights were monitored for the whole length of the treatment. All the experiments have been performed under the EU regulatory standards, within the project number 71/2019-PR authorized by the Ministry of Health, Italy.

## Results

### CAB treatment efficiently reduces cell proliferation in NETs

**Figure 1:**
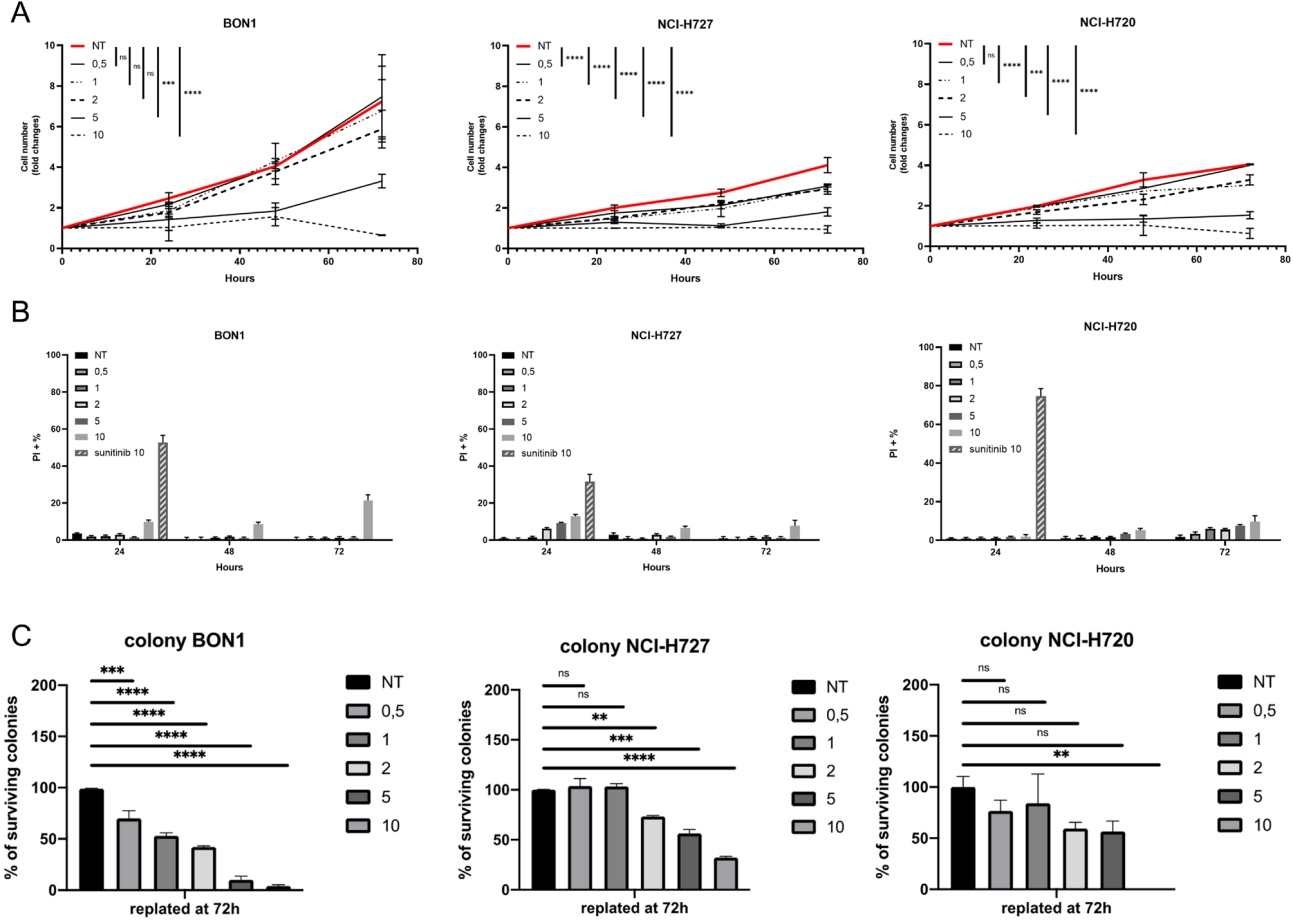
(A) The indicated cell lines were treated (in independent experiments, performed in triplicate) with different doses of cabozantinib (µM), for the indicated times. (B) Percentage of dead cells was measured by exclusion of propidium iodide (PI). Cells were treated as in (A). (C) After 72 h of treatment, cells were plated at low density, and colonies were scored after at least 1 week of incubation (in the absence of the drug). A full-colour version of this figure can be found at https://doi.org/10.1530/ERC-23-0232.

For all cell lines, we observed a statistically significant decrease in viability in the range of clinically relevant concentrations (2–5 µM) with low-to-mild increases in cell death. Cell lines showed a different level of sensitivity: BON1 having an intermediate response, NCI-H727 and NCI-H720 showing the highest sensitivity (Fig. 1A). At 10 µM, all cell lines showed a similarly strong response (Fig. 1A). We then measured the extent of cell death induced by CAB comparing to SUN: interestingly, while high doses of SUN (10 µM) strongly induced cell death in all cell lines, CAB was less active in the induction of cell death. Additionally, the cytotoxic effect was observed for the tested cell lines at the high concentration of 10 µM, with the only exception being that of NCI-H727 cells which showed a low but measurable induction of cell death, even at lower, clinically relevant doses (Fig. 1B). We then explored the clonogenic ability on treated NET cells, to study long-term effects of single-dose treatment (since the incubation at low density to allow for colony formation occurred in the absence of compound). Interestingly, NET cell lines showed a diversified response to treatment with BON1 cells showing a dose-dependent decrease even at low concentrations, NCI-H727 being non-responding at concentrations below 2 µM and NCI-H720 being essentially resistant to treatment (Fig. 1C).

### CAB treatment resulted in cytostatic over cytotoxic effect and polynucleation

**Figure 2:**
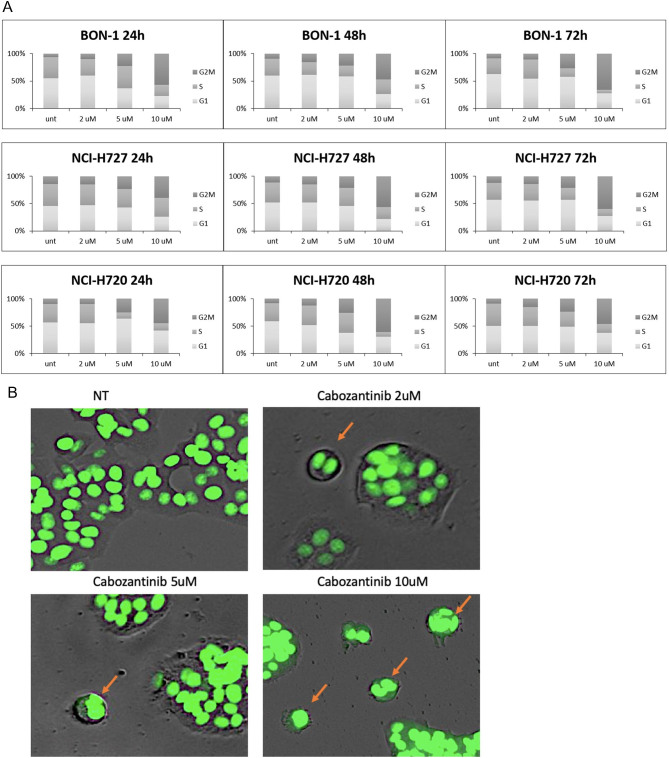
(A) The indicated cell lines were treated (in independent experiments, performed in triplicate) with different doses of cabozantinib (µM), for the indicated times. Distribution of cells in the different phases of cell cycle was measured by propidium iodide. (B) BON1 cells were stained for DNA content: arrows point to cells arrested during mitosis in a polynucleated state. A full-colour version of this figure can be found at https://doi.org/10.1530/ERC-23-0232.

Consistent with the effect on cell viability, we observed a clear, dose-dependent effect on the cell cycle, with an increase of the G2/M fraction which was significant at clinically relevant doses (2–5 µM), and dramatic at 10 µM, with more than 50% of cells in the G2/M phase (Fig. 2A). Two-way ANOVA test has been performed on G2/M values against the untreated control, and the results are shown in Supplementary Table 1 (see section on supplementary materials given at the end of this article). In fact, we noticed several cells arrested during mitosis, resulting in the accumulation of polynucleated cells upon higher doses of CAB already after 24 h of treatment (Fig. 2B). Polynucleated cells have been first excluded from PI cell cycle analysis and separately evaluated as the number of events in FACS that were showing a singlet made by a DNA quantity above 4n (G2). BON1 cells show an endoreplication (aberrant S phase) already starting after 24 h with higher doses of CAB, and strongly visible at 48 h, resulting in the accumulation of polynucleated cells at 72 h (polynuclei). A graph to quantify our observations is represented in Supplementary Fig. 1.

### MCL1 modulation dictates molecular switch between cytostatic and cytotoxic effects in c-MET inhibitor CAB

**Figure 3:**
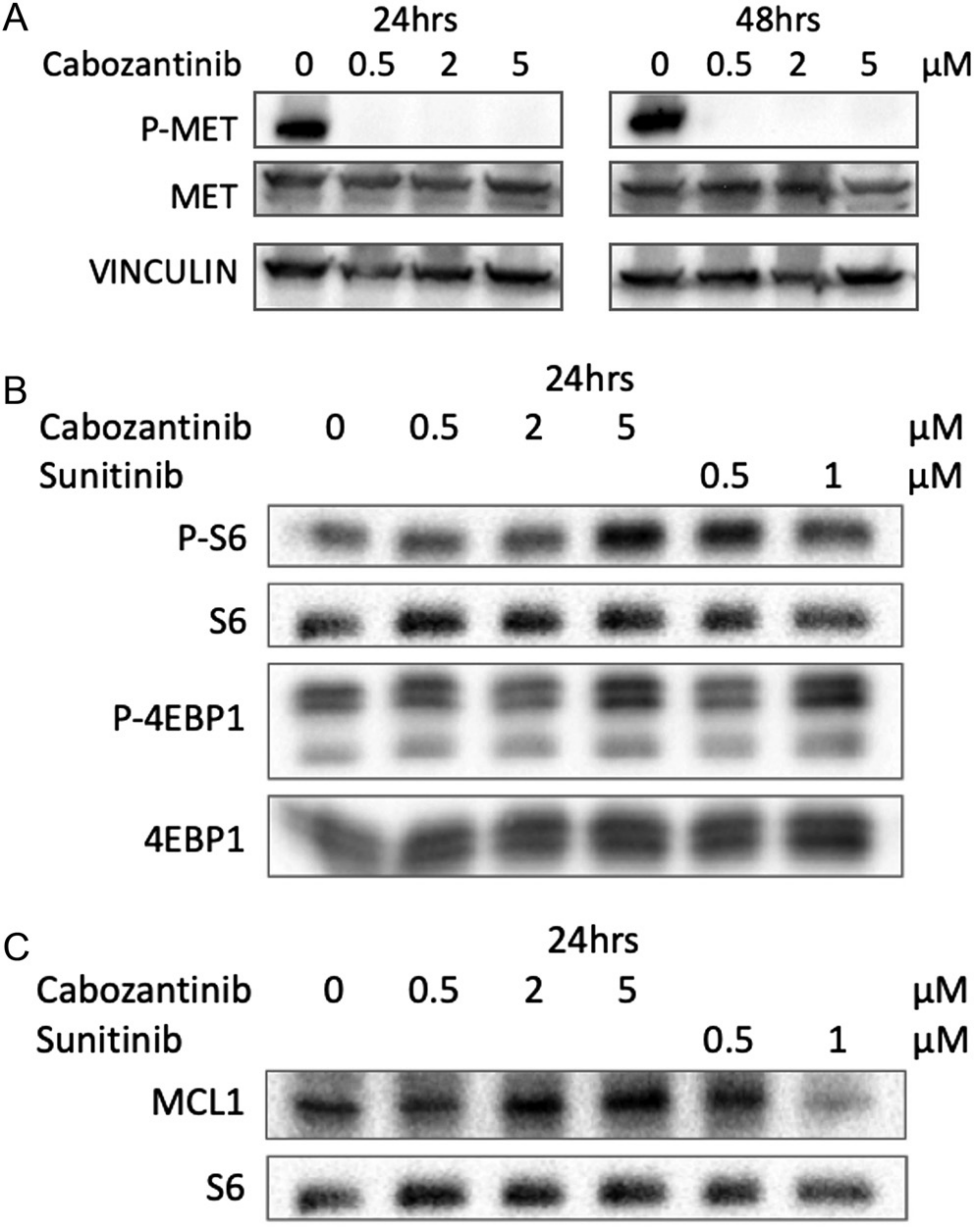
BON1 cells were treated with different doses of cabozantinib or sunitinib (µM), for the indicated times. (A) Analysis of MET phosphorylation. (B) Analysis of mTOR pathway. (C) Analysis of MCL-1 levels.

At the molecular level, we confirmed the activity of CAB on the target, since it showed a complete inhibition of c-MET phosphorylation in BON1 cells already at the lowest concentration of 0.5 µM (Fig. 3A). We analyzed the phosphorylation status of S6 and 4EBP1 as a readout of mTOR activity: we could not detect a significant modulation of the phosphorylation status of these proteins, with a mild and not fully consistent increase only at 5 µM CAB (Fig. 3B). We then tested whether modulation in MCL-1 protein levels was observed during CAB treatment. Interestingly, CAB and SUN showed strikingly different effects on MCL-1 levels, consistent with the different ability to induce cell death: CAB was essentially ineffective in modulating MCL-1 levels, while SUN showed a strong decrease in protein levels (Fig. 3C).

### CAB affects tumor-induced angiogenesis and migratory response in zebrafish models

**Figure 4:**
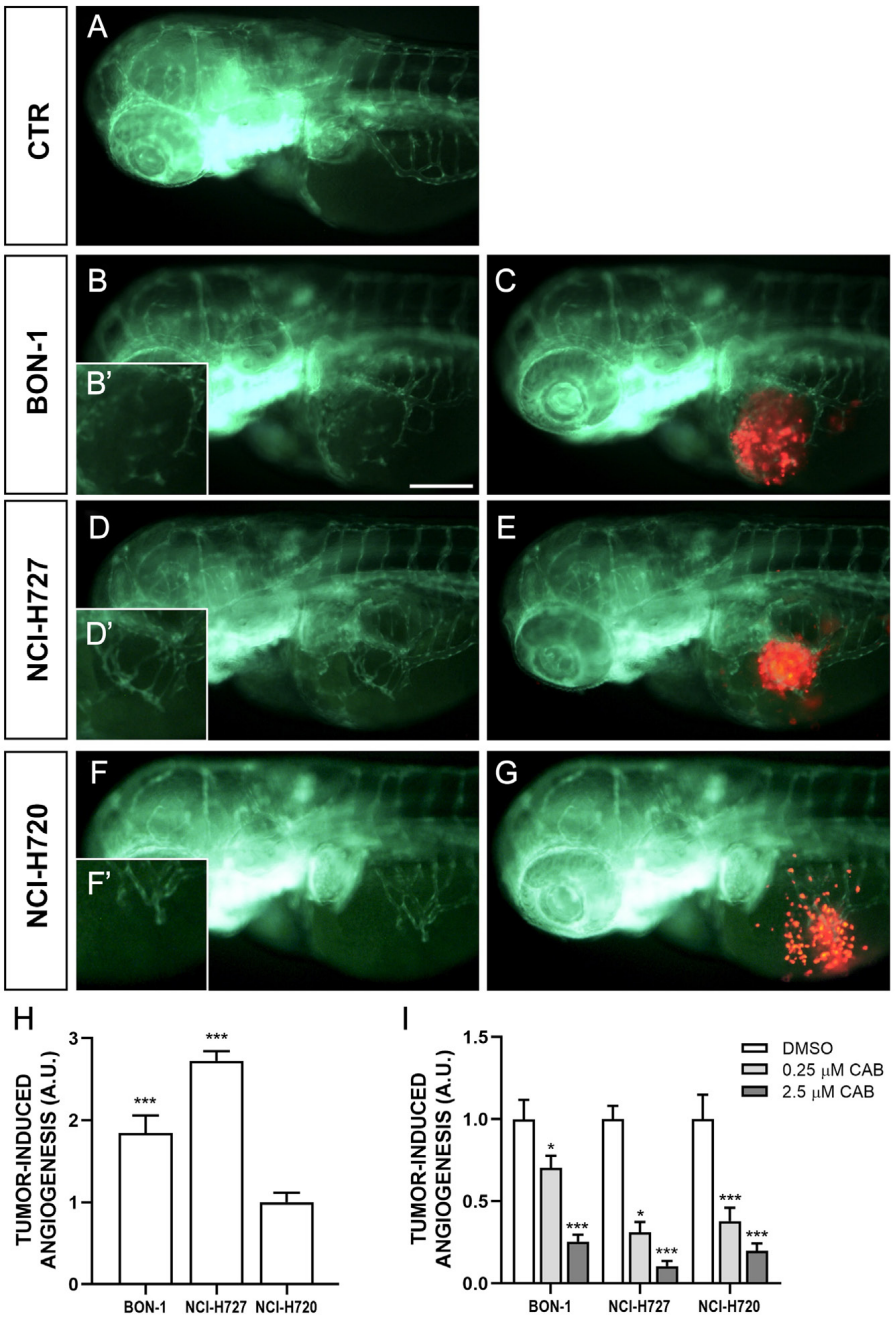
Engraftment of NEN cells in zebrafish embryos. Representative epifluorescence images of *Tg(fli1a:EGFP)^y1^* embryos injected with PBS (control; A) and implanted with red-stained BON-1 cells (B, B’ and C), NCI-H727 cells (D, D’ and E) and NCI-H720 cells (F, F’ and G). Embryos were imaged at 24 h post injection (hpi). The red channel was omitted in panels B, B’, D, D’, F and F’ to facilitate the observation of tumor-induced angiogenesis (green). NEN cells stimulated endothelial sprouting from the SIV plexus within 24 hpi. B’, D’ and F’ are the digital magnification of white-boxed regions. All images are oriented so that rostral is to the left and dorsal is at the top. Scale bar, 100 μm. The graph H showed the quantification of tumor-induced angiogenesis at 24 hpi. NCI-H720 value have been set to 1.0. The graph I showed the quantification of tumor-induced angiogenesis in embryos after 24 h of treatment with DMSO and CAB (0.25 and 2.5 µM). Control (DMSO) values have been set to 1.0. The values reported in the graphs represent the mean ± s.e.m. ^*^P < 0.05;^***^P < 0.001. A full-colour version of this figure can be found at https://doi.org/10.1530/ERC-23-0232.

**Figure 5:**
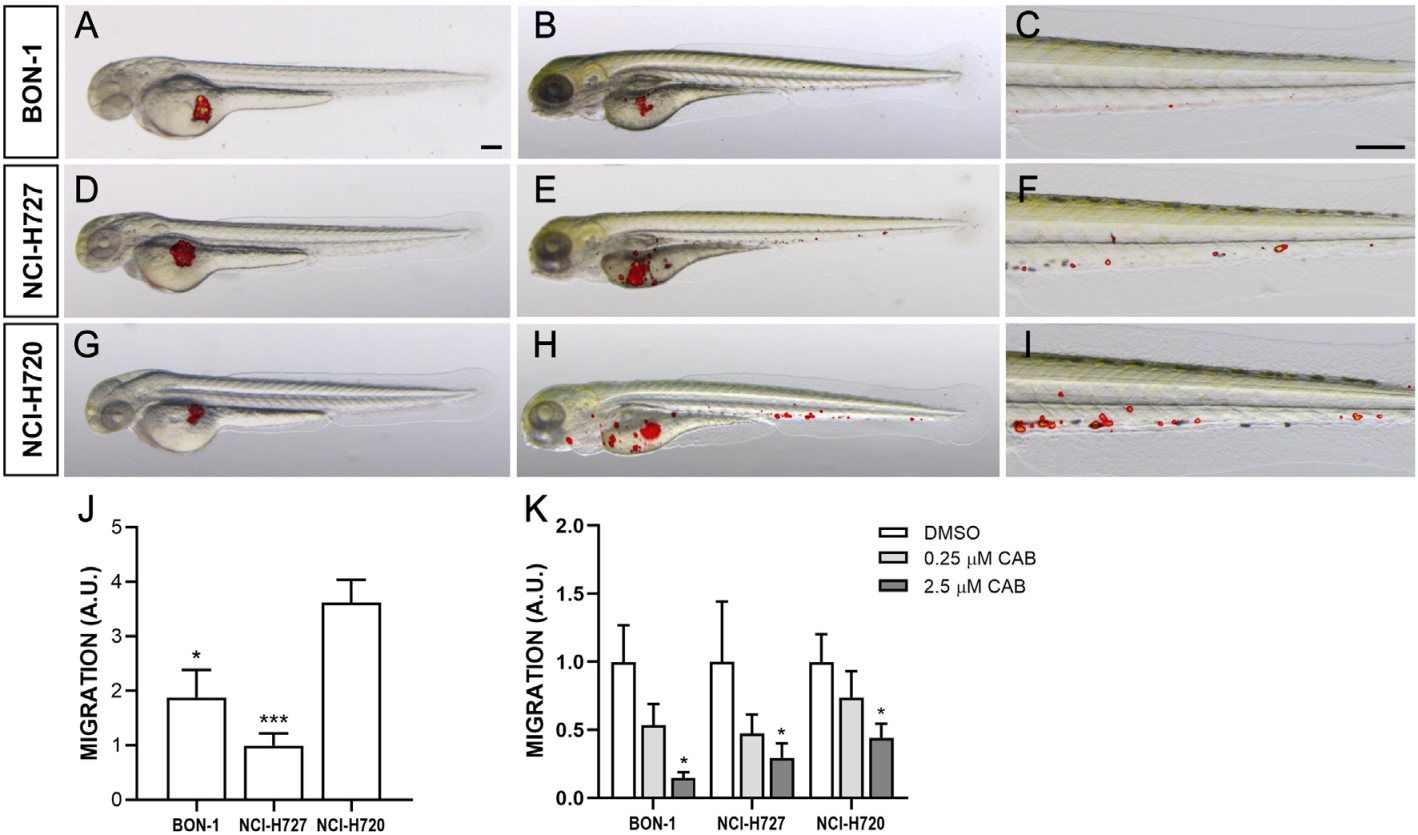
Invasiveness of NEN cells in grafted zebrafish embryos. Overlay of representative fluorescent and bright field images of embryos grafted with red-stained BON-1 (A–C), NCI-H727 (D–F) and NCI-H720 (G–I) cells at 0 (A, D, G) and 48 hpi (B, C, E, F, H, I). For each injected cell line, the tail region was imaged at 48 hpi (C, F, I), showing the spread of NEN cells throughout the embryo body. All images are oriented so that rostral is to the left and dorsal is at the top. Scale bar, 100 μm. The graph J showed the quantification of NEN spread in the tail region at 48 hpi. NCI-H727 value has been set to 1.0. The graph K showed the quantification of NEN spread after 48 h of treatment with DMSO and CAB (0.25 and 2.5 µM). Control (DMSO) values have been set to 1.0. The values reported in the graphs represent the mean ± s.e.m. ^*^P < 0.05; ^**^P < 0.01; ^***^P < 0.001. A full-colour version of this figure can be found at https://doi.org/10.1530/ERC-23-0232.

NCI-H727 and BON-1 cells strongly stimulated angiogenesis in zebrafish embryos, leading to the formation of new endothelial structures, which sprouted from the SIV plexus and CCV toward the tumor implant as early as 24 hpi (Fig. 4). The presence of tumor cells far from the site of injection, in particular along the tail, progressively increased within 48 hpi (Fig. 5). NCI-H720 cells displayed a lower vasoproliferative potential and a higher invasiveness compared to other NET cell lines (Fig. 4I and 5K). Interestingly, CAB drastically inhibited angiogenesis induced by the injection of all NET cell lines in a dose-dependent manner (Fig. 4K). In addition, CAB significantly reduced the number of migrating cells in the tail region (Fig. 5N). In particular, in embryos grafted with all cell lines, CAB significantly inhibited the spreading capability of tumor cells with the highest concentration (2.5 µM) (Fig. 5N).

### CAB induces a modest reduction of tumor growth in xenograft mouse models

**Figure 6:**
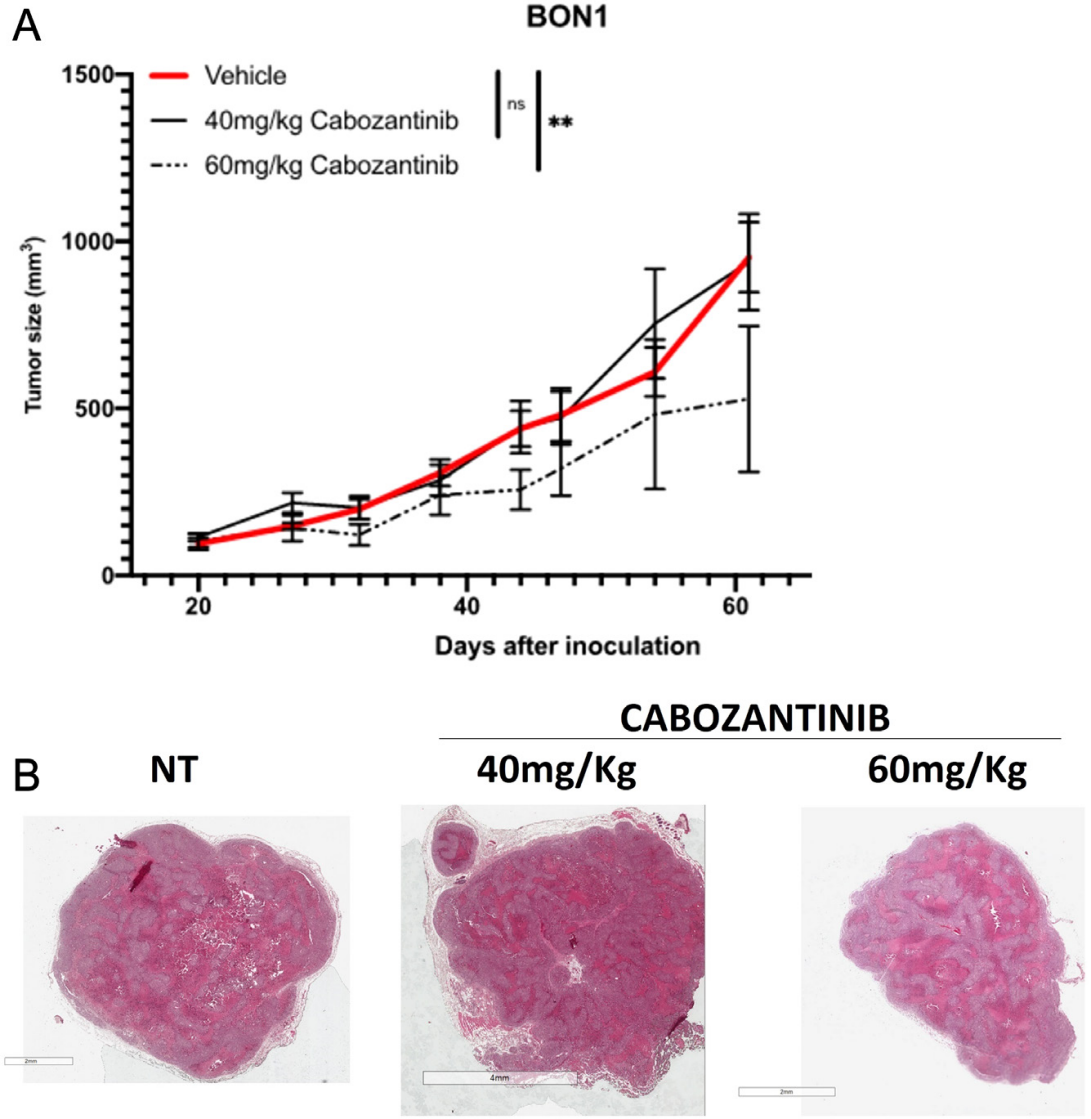
BON1 cells were inoculated into nude mice, and treatment was performed as described in the main text. (A) Tumor size. (B) Representative hematoxylin–eosin staining of tumors at the end of treatment. A full-colour version of this figure can be found at https://doi.org/10.1530/ERC-23-0232.

We tested the effect of CAB *in vivo* in a xenograft model (BON1 cells). Indeed, we observed a reduction in tumor size in the group of 60 mg/kg CAB, while the lower dose was ineffective (*P* < 0.01). Mice weight remained stable during the 3 weeks (Supplementary Fig. 2). BON1 tumors showed areas of necrosis in both untreated and treated samples, without evident differences among treatments (Fig. 6). KI67 and caspase-3 staining also did not show measurable differences among groups, at least at the experimental point tested (end of treatment: Supplementary Fig. 3).

## Discussion and conclusion

We examined the activity of CAB in NET models and investigated the possible mechanism of drug resistance. First, we demonstrated the efficacy of CAB on cell viability in NET cultures (BON-1, NCI-H727 and NCI-H720). We also found a dose-dependent and a time-dependent effect of CAB on cell viability and proliferation alongside a halting of cell cycle progression for endoduplication. Interestingly, we reproduced this effect also in xenograft mouse models despite the reduction of tumor growth* in vivo* was slightly modest. As expected, CAB is also active on its target, downregulating c-MET. Lastly, we observed that CAB drastically affected the vascular network and migration induced by the injection of NET cell lines into zebrafish models, with a similar dose-dependent manner.

Promising compounds with antiangiogenic properties have been developed in the last decades, based on the peculiar hypervascularization of NETs. Among the small molecules under investigation, which are competing to amplify the paradigm treatment in NET, CAB has been under increased attention (Dicitore & Cantone 2022). According to the available preclinical evidence, the antitumor activity of CAB results in a pleiotropic inhibitory effect on different targets (including MET), mainly eliciting an antivascular rather than an antiproliferative effect (Sennino *et al.* 2012). On the other hand, SUN, the only small molecule approved in NETs – precisely for advanced progressive pancreatic NETs (panNETs) – showed both antiproliferative and antiangiogenic properties, but unlike CAB, does not target the MET pathway (Fazio *et al.* 2019). Therefore, given that concurrent inhibition of MET and VEGF signaling mitigates the invasive and metastatic capabilities of panNETs, the dual blockade of both pathways induced by CAB may hypothetically rescue the sensitivity to a prior VEGF treatment.

At the molecular level, we confirmed the activity of CAB on the target, since it showed a complete inhibition of c-MET phosphorylation in BON1 cells already at the lowest concentration of 0.5 µM. However, mechanistic insights about the resistance pathways to CAB still need to be addressed.

In keeping with the concept that SUN exerts its antiproliferative effect through a dual modulation on MCL-1, we investigated the role of the antiapoptotic MCL-1 protein as a potential key regulator of CAB activity (Elgendy *et al.* 2016). In our analysis, CAB was essentially ineffective in down-modulating MCL-1 levels, and this is likely linked to the lack of pro-apoptotic properties. Therefore, the inability to tackle MCL1 could be an important driver of the cytostatic (and not cytotoxic) effect of CAB. One of the proposed resistances to c-MET inhibitors is the modulation of the mTOR pathway that ultimately results in a small portion of tumor cells able to avoid TKI toxicity and regrow *in vitro* (Qi *et al.* 2011). We analyzed the phosphorylation status of S6 and 4EBP1 as a readout of mTOR activity, but we did not detect a significant modulation of the phosphorylation status of these proteins. This leads us to infer that mTOR cannot be clearly ascribed as a resistance pathway involved in the onset of CAB resistance.

The antitumor activity of CAB was further analyzed in a transplantable zebrafish model for NETs, recently developed by Vitale *et al.* (Vitale *et al.* 2014, Gaudenzi *et al.* 2017, Carra & Gaudenzi 2020). We confirmed the suitability and the affordability of zebrafish model for studying tumor angiogenesis and metastatic migration of NET cells under CAB exposure. One of the advantages of this innovative model is that a very limited number of grafted tumor cells in zebrafish embryos can stimulate angiogenesis within a few days and without the need for immunosuppression, because the adaptive immune response is not completely developed during the first month of zebrafish life. Furthermore, unlike mouse models, the transparency of the zebrafish embryos allows investigators to follow the very early steps of invasion, circulation of tumor cells in blood vessels, colonization at secondary organ sites and metastasis formation in real time. Finally, taking into consideration the permeability of the embryo to small molecules dissolved in the fish water, the zebrafish/NET xenograft represents an attractive, fast and technically straightforward platform for drug testing. Interestingly, CAB drastically affected tumor-induced angiogenesis and invasiveness in a dose-dependent manner in our transplantable zebrafish NET model through inhibition of multiple molecular targets, corroborating previous findings reported by Sennino and Reuther (Sennino *et al.* 2012, Reuther *et al.* 2016) in cell cultures and mouse models.

A potential highlight of this study resides in the fact that CAB mainly exerts a cytostatic effect, and this has been proved with both *in vitro* and in mouse models of NET. In our xenograft mouse models, CAB indeed induces a reduction of tumor growth, albeit lower than those observed in prior preclinical studies (Sennino *et al.* 2012). In the work by Sennino *et al.* RipTag2 model has been used to show the increase in invasiveness upon inhibition of VEGF signaling and CAB has been investigated as a multikinase inhibitor to achieve a simultaneous dual inhibition of VEGF and cMet. In the same work, mice have been treated for 3 weeks, during 14–17 weeks, a time which enables RipTag2 mice to spontaneously develop pancreatic tumor. In this context, CAB helped to reduce the volume of vascularization while inhibiting cancer cell motility. In our mouse model, we subcutaneously inoculated tumor cells (5 million cells) and started the treatment when palpable tumor was formed, so when tumor was already established and vascularized, compared to the early stage of development in Sennino *et al.* In other words, the different timing of drug exposure between our experimental model and Sennino’s one accounts for the minor cell death and tumor slowdown observed in our model. This, according to our* in vitro* data, can explain the inability of CAB to activate cell death, while it was extremely effective in reducing vascularization.

The biological effects exerted by CAB together with its inability to down-modulate MCL-1 clearly suggest different known treatments able to downregulate the MCL-1 protein expression (including metabolic drugs such as metformin) to be tested in combination with CAB to increase its efficacy in NETs.

Several ongoing clinical trials are investigating the activity of CAB, as monotherapy or in combination strategies, in different neuroendocrine settings, but – to date – no conclusive results about clinical efficacy and safety have been disclosed (https://clinicaltrials.gov/study/NCT05249114; https://clinicaltrials.gov/study/NCT04427787; https://clinicaltrials.gov/study/NCT05249114;https://clinicaltrials.gov/study/NCT04427787;https://clinicaltrials.gov/study/NCT04412629;https://clinicaltrials.gov/study/NCT04893785; https://clinicaltrials.gov/study/NCT04524208; https://clinicaltrials.gov/study/NCT05289856). Therefore, preclinical and clinical efforts are strongly warranted to provide mechanistic insights intotumor growth and broaden our knowledge in the field of rare tumors.

In conclusion, our results showed a significant dose-dependent and time-dependent effect of CAB in NET cells, more cytostatic than cytotoxic. Interestingly, we reproduced this effect also *in vivo*, since we observed a relatively modest reduction in tumor growth under CAB exposure. We speculate that the inability of CAB to tackle MCL-1 is likely due to the lack of pro-apoptotic properties, but this provides the rationale for a drug combination. Finally, we applied a reliable method for NET preclinical research using a zebrafish/tumor xenograft model for testing *in vivo* the antitumor activity, the antivascular capabilities and the inhibitory effect on cell migration induced by CAB. On this basis, we envisage future research as part of our project to further pursue these promising lines.

## Supplementary Materials

Table S1. Two-way Anova test has been performed on G2/M values against untreated control. Table shows Pvalues, multiple comparisons and statistics referred to panel A fig 2.

Figure S1. (A) Aberrant S phase and polynucleated cells have been quantified by DNA quantification and BrDU incorporation. From G2 phase when nuclei are 4n, a percentage of cells starts to replicate the DNA again observed as BrDU incorporation and increasing DNA quantity above 4n. We defined this as aberrant S phase. Cells ultimately complete aberrant endoreplication of DNA and BrDU is not incorporated anymore, meaning there is no active replication, at this point DNA quantity is double of G2 phase (8n), we defined this as polynucleated cells. (B) example of the analysis.

Figure S2. Mice weight during the 3 weeks treatment. Mice have been weighted before each treatment, from monday to friday and left untouched during the weekend. Weight is expressed in grams (g) and x axis refers to day from the first treatment.

Figure S3. Full set of ki67 e caspase. After 3 weeks of treatment, tumors have been resected, weighted, formaldehyde fixed and then embedded in paraffin blocks. Immunohistochemistry has been performed on 3 randomly selected samples from untreated (NT), CAB 40mg/kg and CAB 60mg/kg. Cuts 4 micrometer thick have been stained for Ki67, Caspases 3 and colored with ematossilin/eosin.

## Declaration of interest

CAC reports personal fees from BMS and Leo Pharma and a research grant from IPSEN (institutional). NF received personal fees from AAA, Hutchinson MediPharma, Merck, Novartis; has financial interest with 4SC, Astellas, Beigene, FIBROGEN, Incyte, IPSEN, MSD and NUCANA; and has received research grant form IPSEN (institutional). FS received personal fees from Ipsen, Novartis, Pfizer, Advanced Accelerator Applications, Merck and has Institutional financial interest with GETNE, Incyte and MSD. SM received research grant from IPSEN (institutional). GV received a research grant from IPSEN (institutional) and funding from Italian Ministry of Health.

## Funding

This work was supported by the IPSEN Company with a research grantand partially supported by the Italian Ministry of Health (IRCCS funding ‘Ricerca Corrente’, ZEBRANET, code: 05C402_2014).

## Data availability

The datasets generated and/or analyzed during the current study are not publicly available but are available from the corresponding author on reasonable request.

## Author contribution statement

Chiara Alessandra Cella: conceptualization, formal analysis, investigation, data curation, writing – original draft. Riccardo Cazzoli: conceptualization, formal analysis, investigation, data curation, writing – original draft. Nicola Fazio: conceptualization, resources, supervision, project administration and funding acquisition. Giuseppina De Petro: conceptualization, data curation, supervision and project administration. Germano Gaudenzi: methodology, formal analysis, investigation and data curation. Silvia Carra: methodology, formal analysis, investigation and data curation. Francesca Spada: conceptualization and manuscript editing. Ilaria Grossi: conceptualization, manuscript editing. Saverio Minucci: conceptualization, resources, supervision, project administration and funding acquisition. Giovanni Vitale: conceptualization, resources, supervision, project administration and funding acquisition.
