# Supplementary material for: Cabozantinib in neuroendocrine tumors: tackling drug activity and resistance mechanisms
Source: Endocr Relat Cancer. 2023 Oct 18;30(12):e230232. doi: 10.1530/ERC-23-0232 (PMC10644769; doi:10.1530/ERC-23-0232)
Supplement: Table S1. Two-way Anova test has been performed on G2/M values against untreated control. Table shows Pvalues, multiple comparisons and statistics referred to panel A fig 2. [file supplementary_table_1.pdf]

BON1			
	**NT vs 2µM CAB**	**NT vs 5µM CAB**	**NT vs 10µM CAB**
**G2 24hrs**	**** Pvalue <0.0001	**** Pvalue <0.0001	**** Pvalue <0.0001
**G2 48hrs**	**** Pvalue <0.0001	**** Pvalue <0.0001	**** Pvalue <0.0001
**G2 72hrs**	*** Pvalue 0.0002	**** Pvalue <0.0001	**** Pvalue <0.0001
**NCI-H727**			
	**NT vs 2µM CAB**	**NT vs 5µM CAB**	**NT vs 10µM CAB**
**G2 24hrs**	ns Pvalue 0.0681	**** Pvalue <0.0001	**** Pvalue <0.0001
**G2 48hrs**	**** Pvalue <0.0001	**** Pvalue <0.0001	**** Pvalue <0.0001
**G2 72hrs**	** Pvalue 0.001	**** Pvalue <0.0001	**** Pvalue <0.0001
**NCI-H720**			
	**NT vs 2µM CAB**	**NT vs 5µM CAB**	**NT vs 10µM CAB**
**G2 24hrs**	ns Pvalue 0.1421	**** Pvalue <0.0001	**** Pvalue <0.0001
**G2 48hrs**	**** Pvalue <0.0001	**** Pvalue <0.0001	**** Pvalue <0.0001
**G2 72hrs**	**** Pvalue <0.0001	**** Pvalue <0.0001	**** Pvalue <0.0001

Table S 1. Two-way Anova test has been performed on G2/M values against untreated control. Table shows Pvalues, multiple comparisons and statistics referred to panel A fig 2.
