# Supplementary figures and images for: Cabozantinib in neuroendocrine tumors: tackling drug activity and resistance mechanisms
Source: Endocr Relat Cancer. 2023 Oct 18;30(12):e230232. doi: 10.1530/ERC-23-0232 (PMC10644769; doi:10.1530/ERC-23-0232)

A)

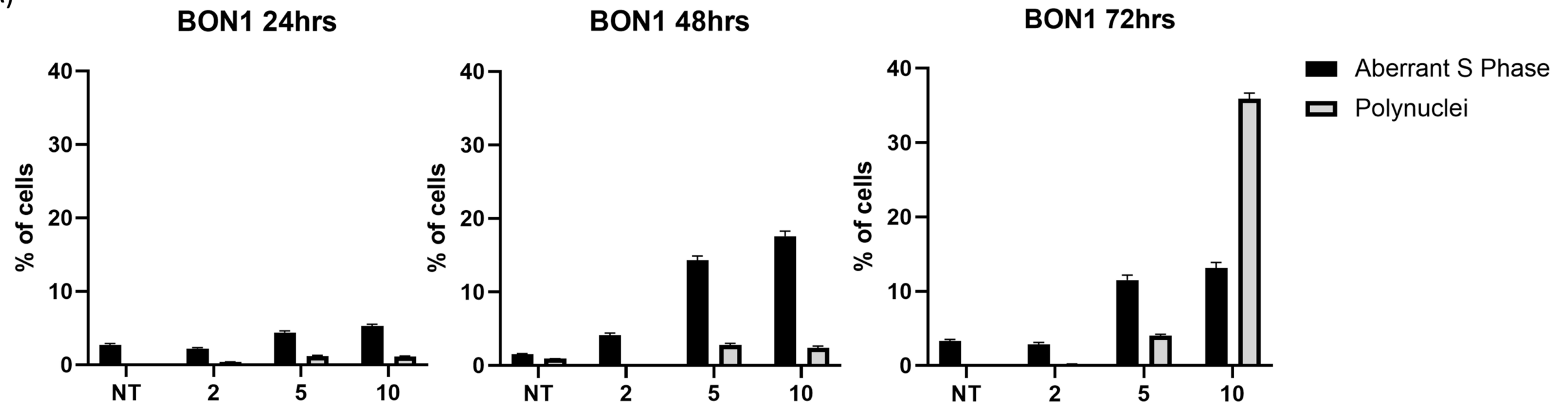

B)

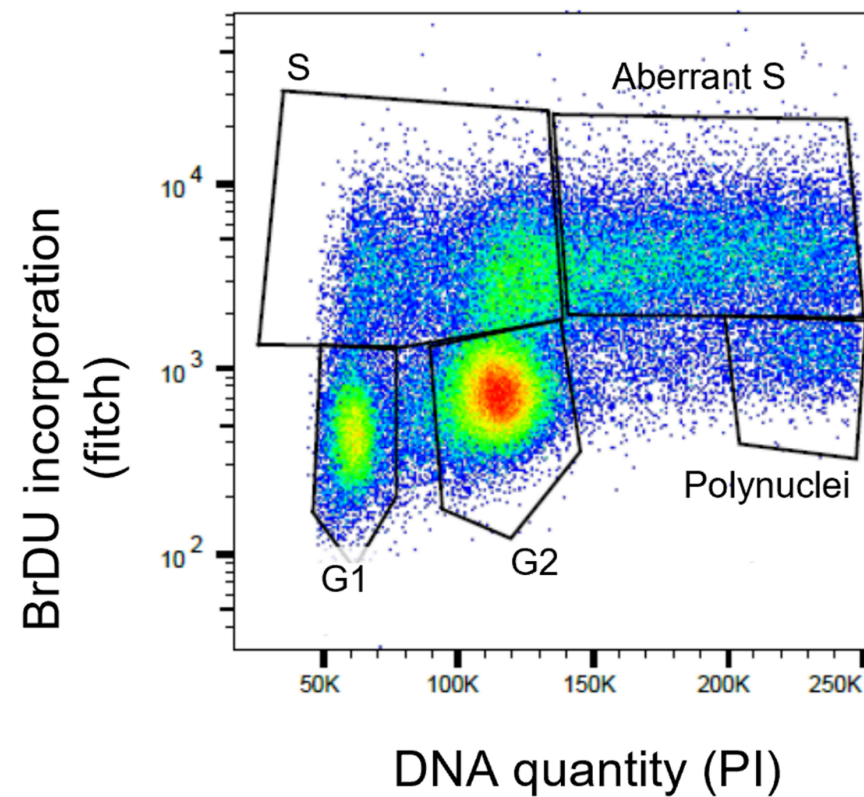

Supplement: Figure S1. (A) Aberrant S phase and polynucleated cells have been quantified by DNA quantification and BrDU incorporation. From G2 phase when nuclei are 4n, a percentage of cells starts to replicate the DNA again observed as BrDU incorporation and increasing DNA quantity above 4n. We defined this as [file supplementary_figure_1.pdf]

# Mice weight

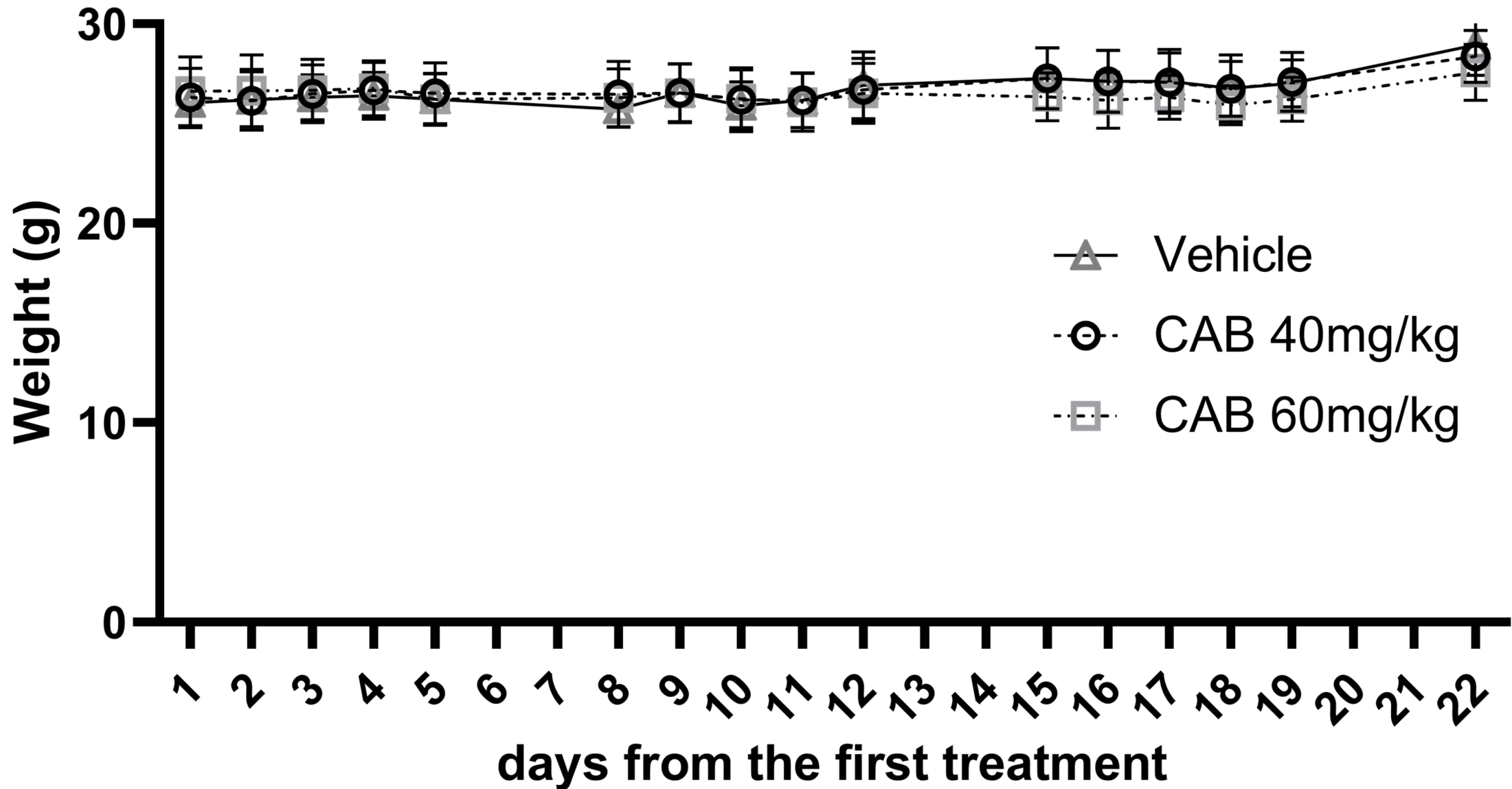

Supplement: Figure S2. Mice weight during the 3 weeks treatment. Mice have been weighted before each treatment, from monday to friday and left untouched during the weekend. Weight is expressed in grams (g) and x axis refers to day from the first treatment. [file supplementary_figure_2.pdf]

## Ematossilin/eosin

## KI67

## Caspase 3

NT

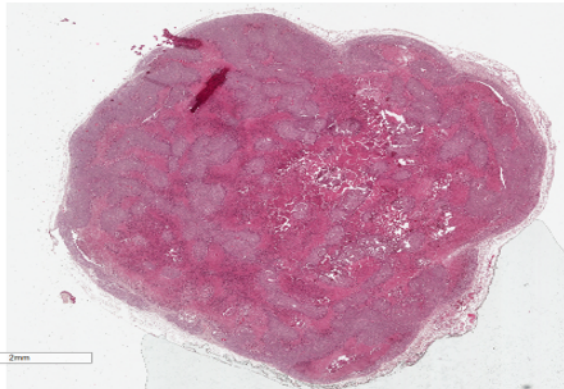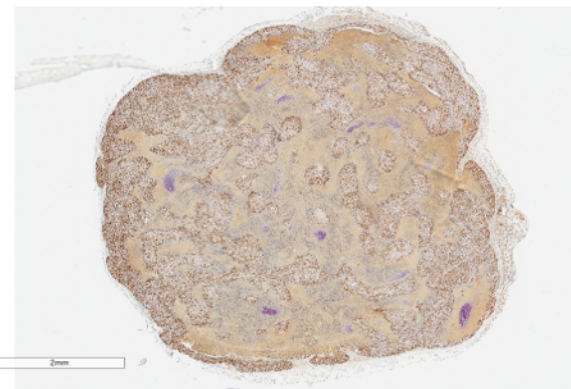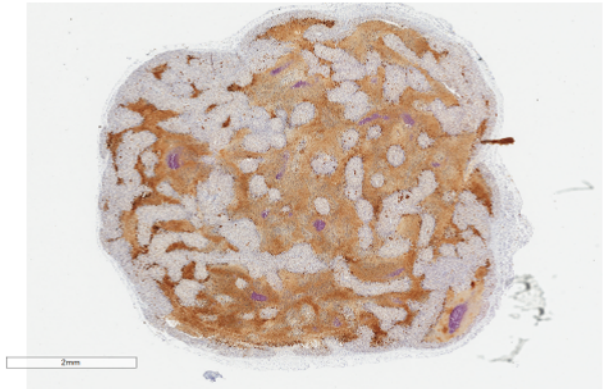

CAB 40  
mg/kg

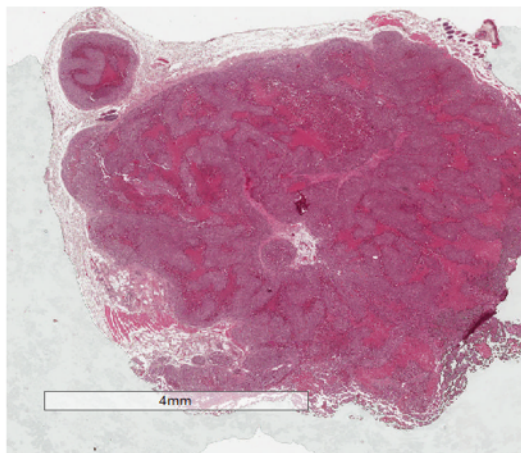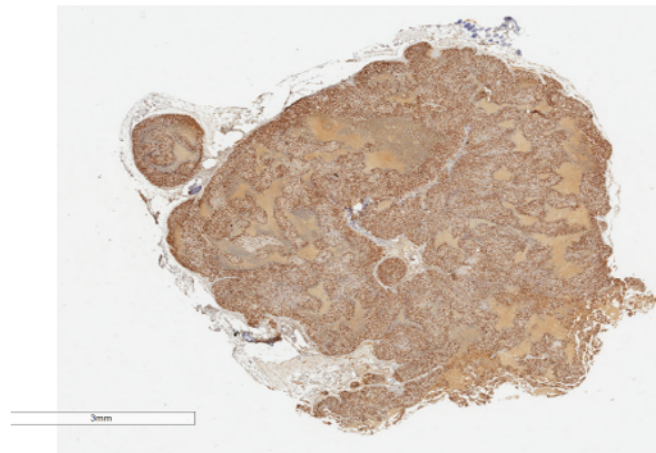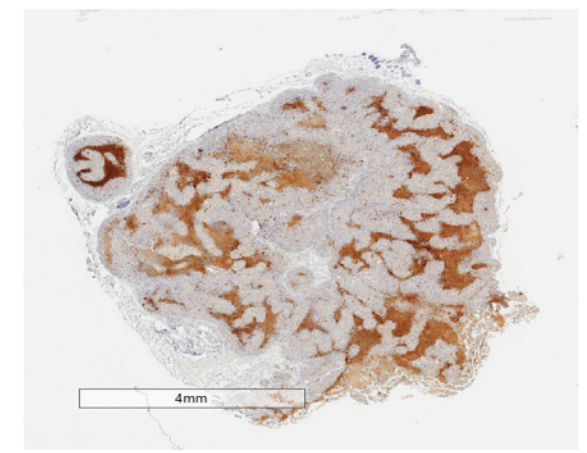

CAB 60  
mg/kg

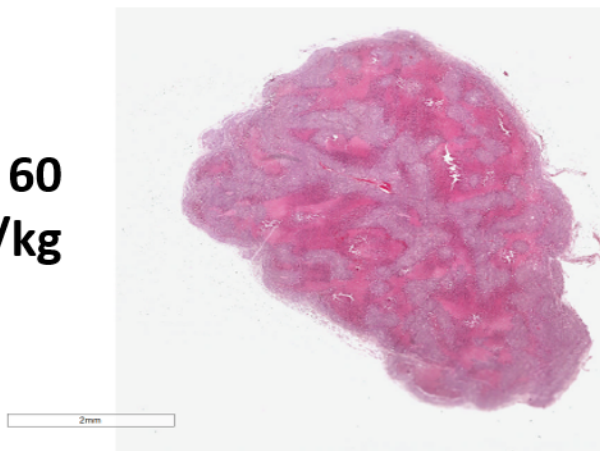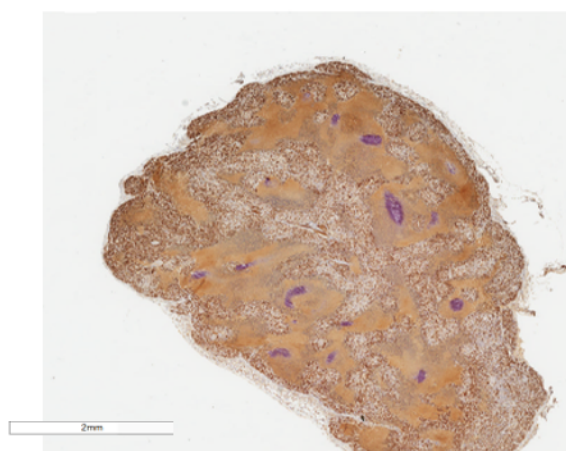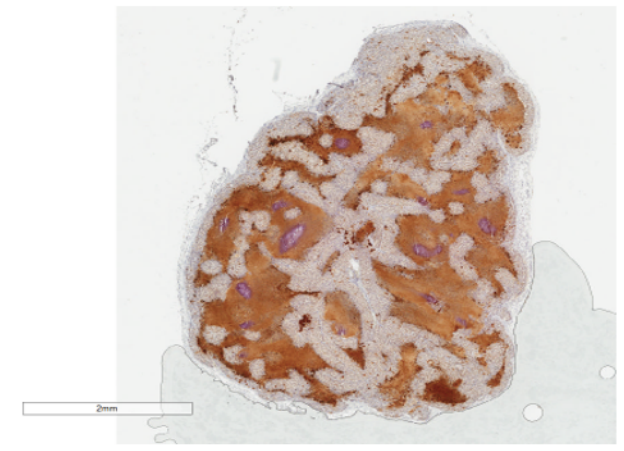

Supplement: Figure S3. Full set of ki67 e caspase. After 3 weeks of treatment, tumors have been resected, weighted, formaldehyde fixed and then embedded in paraffin blocks. Immunohistochemistry has been performed on 3 randomly selected samples from untreated (NT), CAB 40mg/kg and CAB 60mg/kg. Cuts 4 micrometer  [file supplementary_figure_3.pdf]
